# Comprehensive Global Analysis of Future Trends in Artificial Intelligence‐Assisted Veterinary Medicine

**DOI:** 10.1002/vms3.70258

**Published:** 2025-03-27

**Authors:** Sadi Elasan, Osman Yilmaz

**Affiliations:** ^1^ Department of Biostatistics, Faculty of Medicine Van Yuzuncu Yil University Van Türkiye; ^2^ Department of Anatomy, Faculty of Veterinary Medicine Van Yuzuncu Yil University Van Türkiye

**Keywords:** animal health, artificial intelligence, global trends, veterinary medicine

## Abstract

**Background:**

This study conducts a bibliometric analysis of global trends in ‘artificial intelligence studies in veterinary medicine’. The analysis aims to summarise the publications of researchers from various disciplines related to artificial intelligence in veterinary medicine, thereby predicting future trends of AI in this field. The primary objective of the study is to investigate publications pertaining to artificial intelligence in veterinary medicine worldwide and to analyse trends and future developments in this area.

**Methods:**

This bibliometric study examines artificial intelligence research in veterinary medicine conducted worldwide from 1990 to 2024. To achieve this, a search using the keywords ‘artificial intelligence’ and ‘veterinary medicine’ was performed in the Web of Science (WOS) database, resulting in the identification of 1497 studies. After excluding irrelevant publications and those outside the scope of articles, a total of 1400 articles were included in the analysis. The data collection process utilised titles, author names, publication years, journal names, and citation counts. All textual data were analysed using VOSviewer software to ensure accuracy and reliability. In this study, analyses conducted through text mining and data visualisation techniques (e.g., bubble maps) facilitated a clearer understanding of the results.

**Results:**

This study presents information about 1400 articles obtained from the WOS database and a total of 44,700 citations for these articles. The average number of citations per article is 32, with an H‐index of 74. A rapid increase in both the number of articles and citations has been observed since 2019. The majority of the articles (30%) were published in the fields of veterinary sciences, artificial intelligence, and computer sciences. The United States, Taiwan and the United Kingdom are the leading countries, accounting for 84% of the published articles in this field. Additionally, 12% of the articles were published in the area of veterinary sciences, and 85% of the articles fall within the SCI‐Expanded category.

**Conclusions:**

The findings of our study indicate that there are numerous active researchers in the field of artificial intelligence in veterinary medicine and that research in this area is steadily increasing. This bibliometric analysis highlights global trends and significant works in artificial intelligence within veterinary medicine, providing valuable insights into the future directions of research in this field. As the analysis aims solely to identify trends and patterns in the literature, it does not intend to evaluate the applicability of the subject matter.

**Highlights:**

1. *Analysis of Global Trends*: This study comprehensively analyses the global trends and effects of research on artificial intelligence in veterinary medicine. In this context, it contributes to the identification of significant changes and developments in the literature.
2. *Rapidly Spreading Research*: Research on artificial intelligence in veterinary medicine has rapidly expanded in recent years, and this trend is expected to continue. The increase in studies indicates an expansion of knowledge and applications in this field.
3. *Diagnostic and Therapeutic Tools*: Artificial intelligence research serves as a valuable tool in veterinary medicine, particularly in improving the diagnosis and treatment processes for various diseases. This contributes to the development of more effective methods for animal health and care.
4. *Increasing Number of Publications*: The number of studies on artificial intelligence in veterinary medicine worldwide is increasing each year. Notably, after the Covid‐19 pandemic, there has been a significant rise in publications in this field. This indicates that the importance of artificial intelligence in both human and animal health has grown, with the pandemic intensifying research interest.
5. *Prominent Countries*: Among the countries examined in the study, the United States, Taiwan, England, and Germany emerged as leaders in this research area. Conversely, it was noted that some countries have very few or no academic publications in the field of artificial intelligence in veterinary medicine.

## Graphical abstract

This study demonstrates a global increase in scientific research and publications on artificial intelligence (AI) in veterinary medicine. The analysis aims to summarise the publications of researchers from various disciplines related to AI in veterinary medicine, thereby predicting future trends of AI in this field. Furthermore, identifying key journals, authors, and notable studies points to potential areas for increased use of animal models in AI research in the future. The findings of our study indicate that there are numerous active researchers in the field of AI in veterinary medicine and that research in this area is steadily increasing. In conclusion, a better understanding of the potential of AI in veterinary medicine offers significant opportunities for improving animal health and welfare in the future.

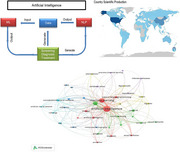

## 1 Introduction

This research includes a comprehensive bibliometric study examining the trends in the use of artificial intelligence (AI) in veterinary medicine worldwide. The applications of artificial intelligence in veterinary medicine are considered to be among the most important technologies shaping the future of modern healthcare. AI provides major innovations in both human and veterinary medicine, bringing radical changes in various areas, from disease diagnosis to treatment processes. Particularly in animal health and treatment, the advantages offered by AI enable veterinarians to make faster, more precise, and more effective decisions.

This study aims to examine the future effects of artificial intelligence in veterinary medicine in light of scientific research and to reveal how these innovations are reflected in the scientific literature. AI introduces significant innovations in both medical and veterinary practices, offering many advantages by transforming animal health and treatment processes. Veterinarians can utilise AI across various fields to expedite diagnosis, prognosis, and treatment, achieving more accurate results and optimising treatment plans. By providing these services, AI makes a considerable contribution to both animal and human health as well as general veterinary practices (Hamet and Tremblay 2017; Jiang et al. 2017).

However, artificial intelligence technologies provide a significant transformation in a wide range of areas such as veterinary anatomy, education, diagnosis and surgical applications. The potential and limitations of artificial intelligence on animal studies bring new opportunities, especially in the analysis of complex anatomical structures and other areas of veterinary medicine. In addition, the use of artificial intelligence‐supported techniques in veterinary medical imaging provides significant contributions to improving clinical outcomes by increasing accuracy, speed, efficiency and data security in diagnostic and surgical processes (Choudhary et al. 2023; Choudhary et al. 2024; Vickram et al. 2024; Yılmaz 2024).

**FIGURE 1:**
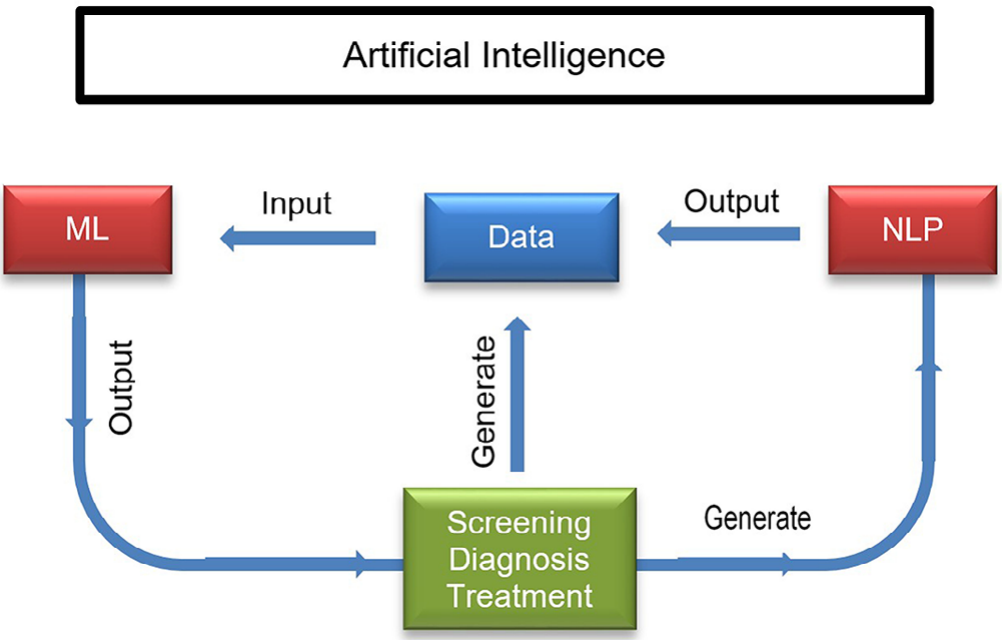
A comprehensive roadmap illustrating the journey from clinical data generation to data enrichment using natural language processing (NLP), advanced data analysis with machine learning (ML), and integration into clinical decision‐making processes.

The AI process follows a pathway that begins with clinical data collection, progresses through data enrichment with Natural Language Processing (NLP), continues with data analysis using Machine Learning (ML), and culminates in the clinical decision‐making process. This roadmap starts and ends with clinical processes. Regardless of how advanced AI techniques may be, they should be inspired by clinical problems and ultimately employed to support clinical applications. Notably, a significant portion of the AI literature focuses on diagnostic imaging, genetic tests, and electrodiagnostic data during the diagnosis stage (Figure 1). For instance, Jha and Topol (2016) encouraged radiologists to leverage AI technologies when analysing diagnostic images containing large amounts of data.

AI‐supported image processing and analysis systems enable veterinarians to interpret medical images, such as X‐rays, ultrasounds, computed tomography (CT), and magnetic resonance imaging (MRI), more quickly and accurately. For instance, these technologies can facilitate the early diagnosis of conditions such as hip dysplasia in dogs or tumours in cats. Consequently, AI‐supported technologies accelerate the diagnostic processes for veterinarians, allowing them to initiate treatment for animals more swiftly and accurately (Hennessey et al. 2022).

Moreover, AI technologies analyse large datasets, consolidating critical information that aids in disease diagnosis, including anamnesis, symptoms, and laboratory results. This capability facilitates the early diagnosis of complex diseases and enables a prompt start to the treatment process (AlZubi 2023). Additionally, these technologies can offer personalised treatment methods by enabling real‐time, individual monitoring of animal health, thereby enhancing the effectiveness of treatment plans (Albadrani et al. 2024; Appleby and Basran 2022).

Through AI analyses, the capacity for rapid big data analysis in the development of new veterinary drugs and the design of clinical trials has accelerated the discovery and testing of potential drug candidates. This capability significantly shortens the time required for drugs to reach the market (Kumar et al. 2023; Paul et al. 2021). Furthermore, various AI‐supported applications provide veterinarians with the opportunity to offer remote consultancy services, which can be crucial in emergencies or when access to veterinary services is challenging (Abu‐Seida et al. 2024).

Work processes in veterinary clinics can be complex and demanding, as patients require effective monitoring, optimised treatment processes, and ensured customer satisfaction simultaneously. This is precisely where innovative technologies like artificial intelligence become invaluable. AI in veterinary medicine serves as a powerful tool to enhance clinic efficiency, improve service quality, and ensure better patient care. Today, the utilisation of artificial intelligence is not merely a preference but a necessity for veterinarians (Tuvay and Ermetin 2023)

This bibliometric study aims to examine the extent to which artificial intelligence in veterinary medicine is represented in global research, the trends in this field, and the prominent topics. Our research seeks to evaluate the current knowledge in this area by analysing the global distribution of scientific studies published on artificial intelligence in veterinary medicine, their impact factors, and the most cited studies. This analysis will serve as an essential guide for understanding both the future of artificial intelligence in veterinary medicine and the acceptance of this technology within the scientific community. Given the rapid advancement of AI technologies in these fields, it is anticipated that AI applications in veterinary medicine will become increasingly prominent in the future, leading to an enhancement in the quality of health services.

## 2 Materials and Methods

To ensure the reliability of this study and the accuracy of the results, a systematic data collection method, a detailed search strategy, and effective network analysis software were employed. The meticulous application of these methods facilitated the collection and analysis of the most current and comprehensive data in the literature. Additionally, the analysis of these data provides the scientific community with valuable insights into current trends and tendencies regarding the future and impact of artificial intelligence studies in veterinary medicine. Global publication trends in AI within veterinary medicine were determined by examining various factors such as the most influential researchers, countries, and the most frequently used keywords.

### 2.1 Data Collection Method and Search Strategy

**FIGURE 2:**
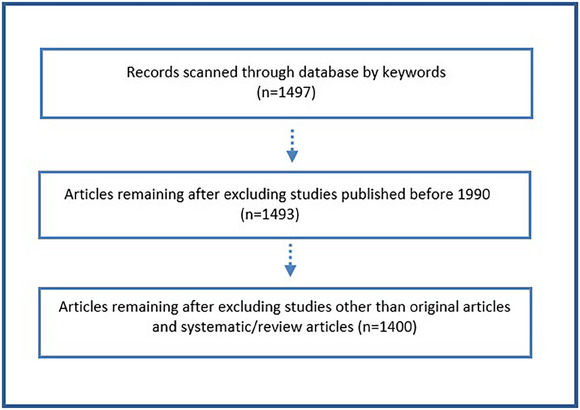
Flow chart of inclusion and exclusion criteria. The scheme explains how the scope of research is determined by presenting in detail the stages of screening, evaluation and final selection.

In this bibliometric study, research on the future and effects of artificial intelligence in veterinary medicine conducted between 1990 and 2024 (last access date: 5 November 2024) was examined using the ‘Web of Science Core Collection (WOS, Clarivate Analytics, Philadelphia, PA, USA)’ database. The search results using the keywords ‘artificial intelligence’ and ‘veterinary medicine’ yielded 1497 studies. Studies published before 1990 were not included in the study. Additionally, 1400 articles were evaluated after eliminating studies that were not original articles, systematic reviews, or review articles. Studies published up to November 2024 were included (Figure 2). The articles in the database were analysed using essential information such as the article title, authors' names, publication year, journal name, and the number of citations. Access to the obtained materials was facilitated through the online library and digital resources of Van Yuzuncu Yıl University. The search process was conducted in English, which is the predominant language in scientific literature.

In the study, publications on global trends in AI studies in veterinary medicine were meticulously examined using bibliometric methods through the WOS database. WOS is a comprehensive database that includes academic articles published across many disciplines and subjects, providing a valuable resource for interdisciplinary research. In this study, publications in the WOS database were systematically collected using specific search terms and subsequently subjected to bibliometric analysis. During the data collection phase, various parameters such as publication growth, the most active countries and research institutions, and keyword matches were analysed in detail. All articles were rigorously reviewed for compliance with the specified criteria.

### 2.2 Network Analysis

In this bibliometric study, ‘VOSviewer (version 1.6.20, Leiden University, Holland)’ and ‘Biblioshiny (version 2.0), R‐Studio (R version 4.2.2)’ software was utilised to determine global trends in AI studies in veterinary medicine and identify key research topics in this field. VOSviewer served as a powerful tool for data visualisation, allowing for detailed analyses of collaboration networks, research trends, and future research topics (Van Eck and Waltman 2022; VOSviewer 2023). Bibliometrix and Biblioshiny, which are open‐source research software running in the R language environment, provide an interactive platform that enables users to perform bibliometric and visual analyses. This platform effectively displays bibliometric data such as publication volume, number of articles, number of citations, and keywords.

The analyses offer a comprehensive perspective on the development of AI applications in veterinary medicine by presenting diagrams and maps that illustrate critical research points, research status, and publication dynamics over time (Aria and Cuccurullo 2017). The WOS database utilised for systematic data collection has provided a robust foundation for analysing scientific publications worldwide. Descriptive statistical techniques and data visualisation methods, such as bubble maps, were employed to present the data obtained from scientific publications. These methods enhance the accuracy and reliability of the research and facilitate a better interpretation of the results.

### 2.3 Bubble Maps

In the bibliometric analyses performed with VOSviewer, graphs known as ‘bubble maps’ depict the grouping of articles published in a research area according to their frequency. These graphs represent each keyword or research group with a ‘bubble’, the size of which varies based on the publication frequency of the relevant keyword or group. The bubbles are colour‐coded, ensuring that related keywords or topics are positioned close to one another. This visualisation method enables users to comprehend datasets more easily and observe research trends more clearly.

## 3 Results

In this study, a total of 1400 articles obtained from the ‘Web of Science Core Collection’ (WOS) database were examined. A total of 44,700 citations were attributed to these articles; when self‐citations are excluded, the remaining number of citations is 44,026. This data plays a crucial role in reflecting the impact and visibility of the articles within the scientific community. The analysis determined that the average number of citations per article was 32. This value indicates that the examined publications are considered valuable by scientists and academics in the research field. The H‐index was calculated to be 74. The H‐index is an important metric used to evaluate the productivity and impact level of a researcher or research group; this high value suggests a significant overall quality and impact of research on artificial intelligence in veterinary medicine.

**FIGURE 3:**
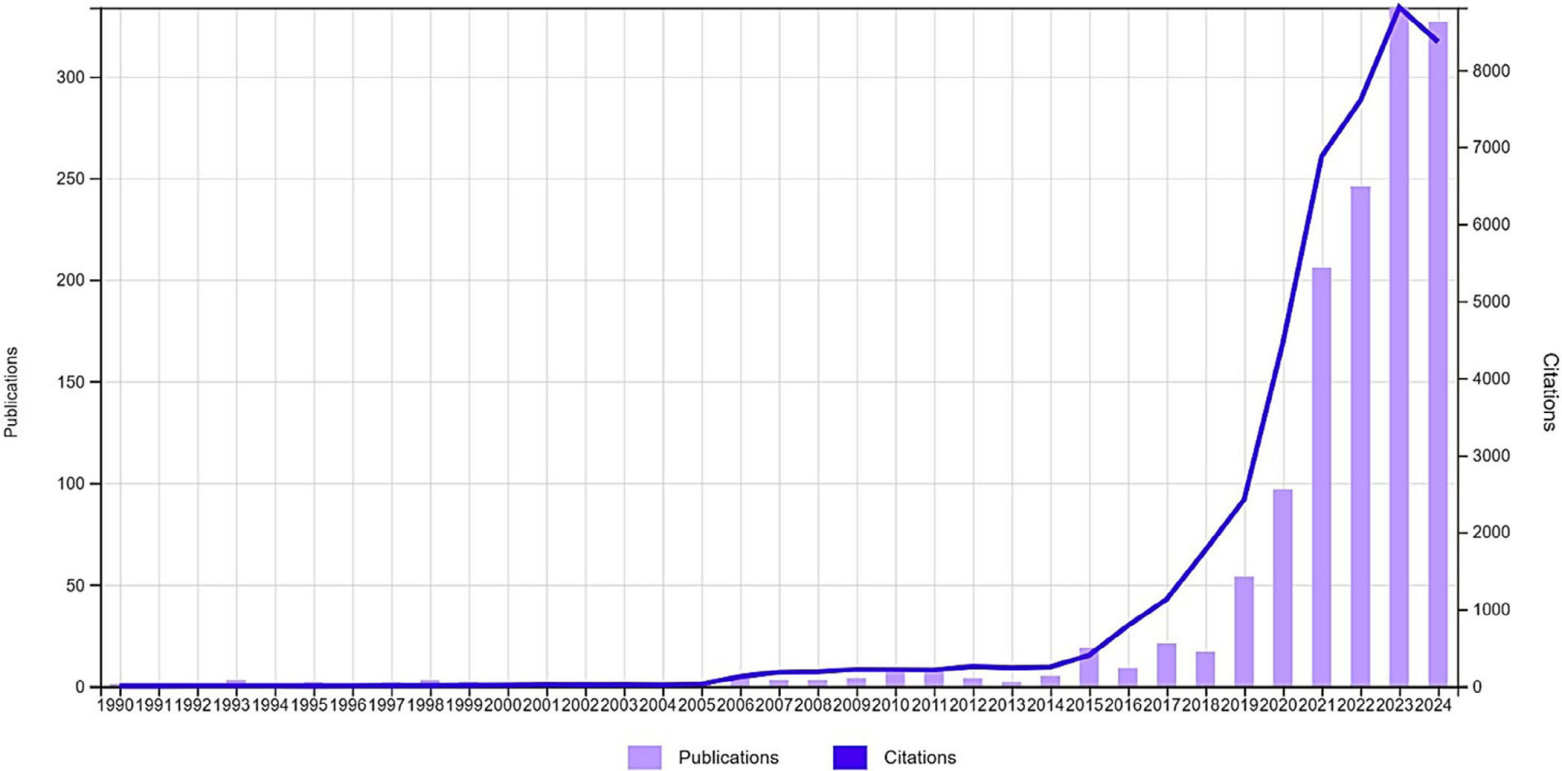
Annual distribution of publications alongside their respective citation frequencies, providing a visual representation of the trends in academic output and scholarly impact over time. The data reflects the evolving prominence of the topic and is based on the latest available figures as of 05.11.2024.

When examining the distribution of published articles over time, it is observed that the number of articles published between 1990 and 2015 was almost negligible. However, a limited number of articles were published between 2006 and 2018. Notably, since 2019, there has been a significant increase in both the number of articles and the number of citations. This increase can be attributed to the growing recognition of the importance of artificial intelligence in the field of veterinary medicine and the corresponding rise in research on this topic. The distribution of publications and citations by year is presented in detail in Figure 3, which clearly visualises these trends.

The analyses conducted indicate that the increasing interest in artificial intelligence applications in veterinary medicine contributes to enhanced research in this field, thereby expanding the body of scientific knowledge. These findings provide a foundation that will facilitate further diversification and deepening of studies on this subject in the future.

In our research, analyses conducted in the context of the selected keywords aimed to determine the broadest and most interconnected subject areas of artificial intelligence in veterinary medicine. In this regard, it was identified that keywords such as ‘artificial intelligence’, ‘deep learning’, ‘convolutional neural network’, ‘dog’, ‘Covid‐19’, ‘radiology’ and ‘disease’ stood out. These keywords reflect the diversity and scope of research regarding the applicability and effects of artificial intelligence in veterinary medicine.

In particular, the terms ‘artificial intelligence’ and ‘deep learning’ are frequently encountered in the literature as fundamental components and techniques of artificial intelligence. The term ‘convolutional neural network’ (CNN) is a deep learning model often utilised in the analysis of visual data, constituting an essential part of studies evaluating medical images such as X‐rays and ultrasounds in veterinary medicine. The presence of the keyword ‘dog’ indicates significant interest in health research and applications, particularly concerning canine health. This highlights the impact of artificial intelligence applications in veterinary medicine on animal health.

The term ‘Covid‐19’ emerges as a factor that enhances the significance of artificial intelligence in veterinary medicine during the pandemic. The use of artificial intelligence applications in animal health management and disease prevention has guided research during this period. The keywords ‘radiology’ and ‘disease’ underscore the importance of AI‐supported diagnostic processes and disease management in veterinary medicine. The connections between these keywords illustrate the focal areas of research and highlight the topics receiving greater attention.

**FIGURE 4:**
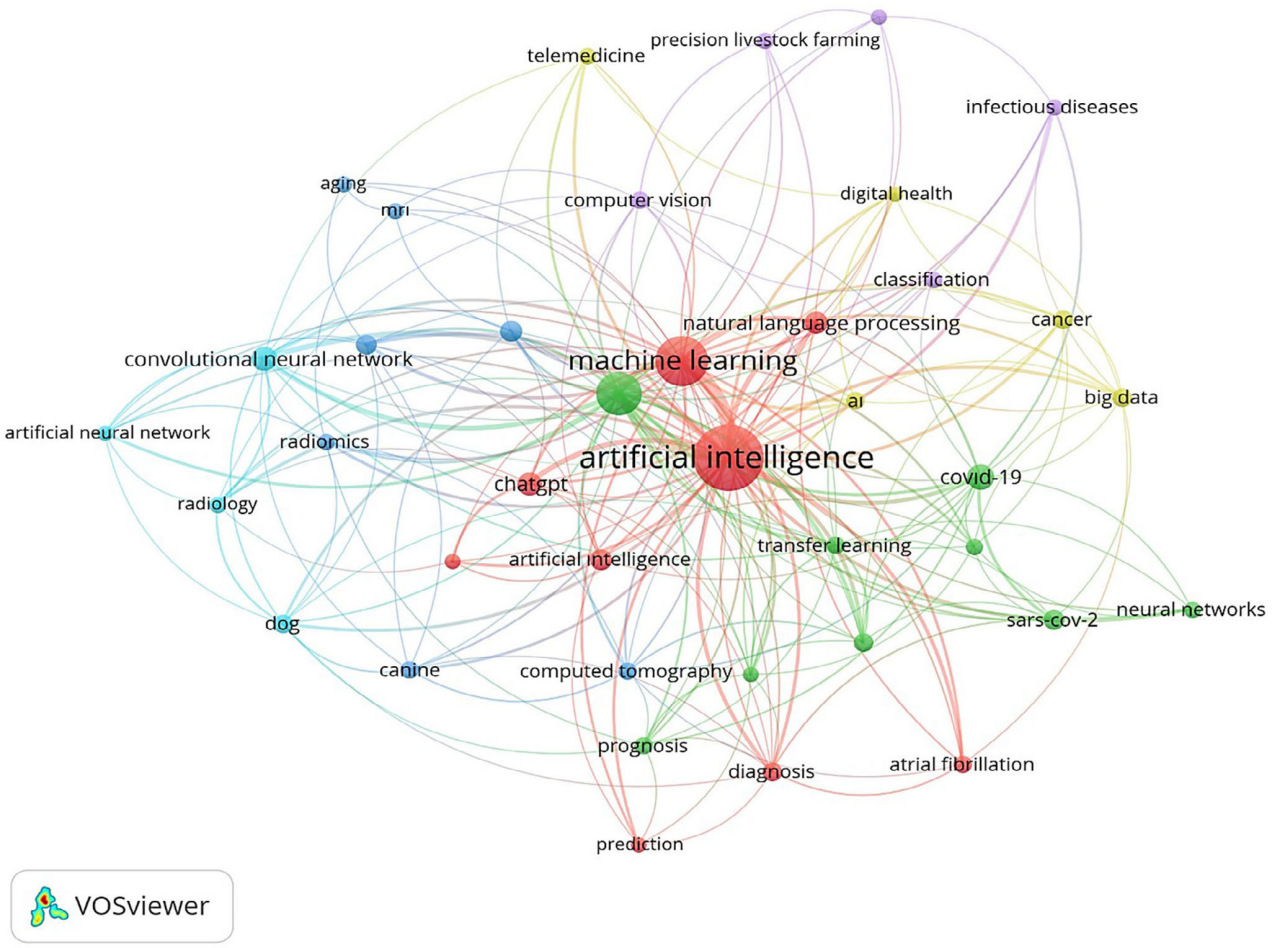
Keyword analysis illustrating the connection between the central topic and a range of specific keywords, along with the frequency of their usage in relevant publications. This analysis provides a deeper understanding of how certain terms are linked to the topic and how their prominence has varied over time.

**FIGURE 5:**
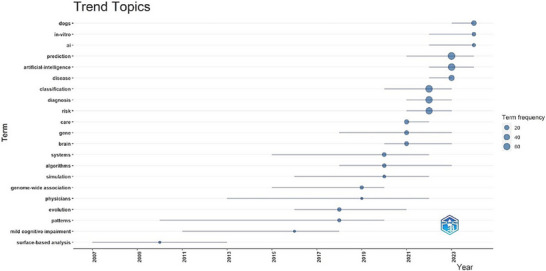
Trend terms analysis presented through a graph, showcasing the evolution of specific terms over time. This analysis highlights the shifting focus of research, demonstrating how the frequency of certain terms has changed year by year.

**FIGURE 6:**
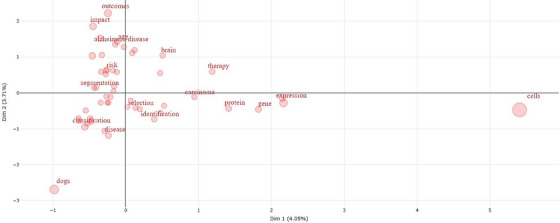
Distribution of research topics across various dimensions, providing a visual representation of how different aspects of the topic are explored within the literature. This distribution illustrates the breadth and diversity of research focus, categorised by key dimensions.

In this context, Figures 4, 5, 6 present graphs that visualise the relationships between the keywords and the effectiveness of these relationships in the research area. These graphs reveal the frequency and connectivity levels of the relevant keywords, aiding our understanding of the current trends in artificial intelligence studies in veterinary medicine.

When examining the distribution of articles on artificial intelligence in veterinary medicine by field, it is observed that the highest percentages are concentrated in specific disciplines. Among the most published fields, Veterinary Sciences stands out with 11.8%, followed closely by Computer Science at 11.6%, Science Technology Other Topics at 8.1%, and Engineering at 7.5%. These percentages highlight the importance of artificial intelligence applications in veterinary medicine and related branches of science. Notably, Veterinary Sciences emerged as the field where artificial intelligence studies are predominantly conducted. However, the contribution of this field to the total number of articles remains at a modest rate of 11%. This situation suggests that the potential of artificial intelligence in veterinary medicine has not yet been fully realised and that further research is warranted in this area.

Table 1 provides a detailed comparison of the number and rates of publications across different disciplines, facilitating comparisons among these fields. This table serves as a valuable resource, particularly in identifying which disciplines contribute to veterinary medicine through artificial intelligence and which areas warrant greater focus.

**TABLE 1:** Categories of publications.

Research areas	Record count	% of 1400
Veterinary Sciences	165	11.79
Computer Science	163	11.64
Science Technology Other Topics	114	8.14
Engineering	105	7.50
General Internal Medicine	105	7.50
Neurosciences Neurology	100	7.14
Agriculture	76	5.43
Medical Informatics	73	5.21
Radiology Nuclear Medical Imaging	68	4.86
Biochemistry Molecular Biology	67	4.79
Oncology	60	4.29
Health Care Sciences Services	54	3.86
Cardiovascular System Cardiology	53	3.79
Research Experimental Medicine	53	3.79
Chemistry	49	3.50

*Note*: Showing 15 out of 96 entries.

In conclusion, the concentration of artificial intelligence applications in veterinary medicine within only a few disciplines indicates that future research in this domain should be broadened, and more interdisciplinary collaboration should be encouraged. Increased support for these areas will enable a more effective utilisation of artificial intelligence technologies in veterinary medicine.

The search results using the keywords ‘artificial intelligence’ and ‘veterinary medicine’ yielded 1497 studies. Studies published before 1990 were not included in the study. Additionally, 1400 articles were evaluated after eliminating studies that were not original articles, systematic reviews, or review articles. Studies published up to November 2024 were included (Table 1).

**FIGURE 7:**
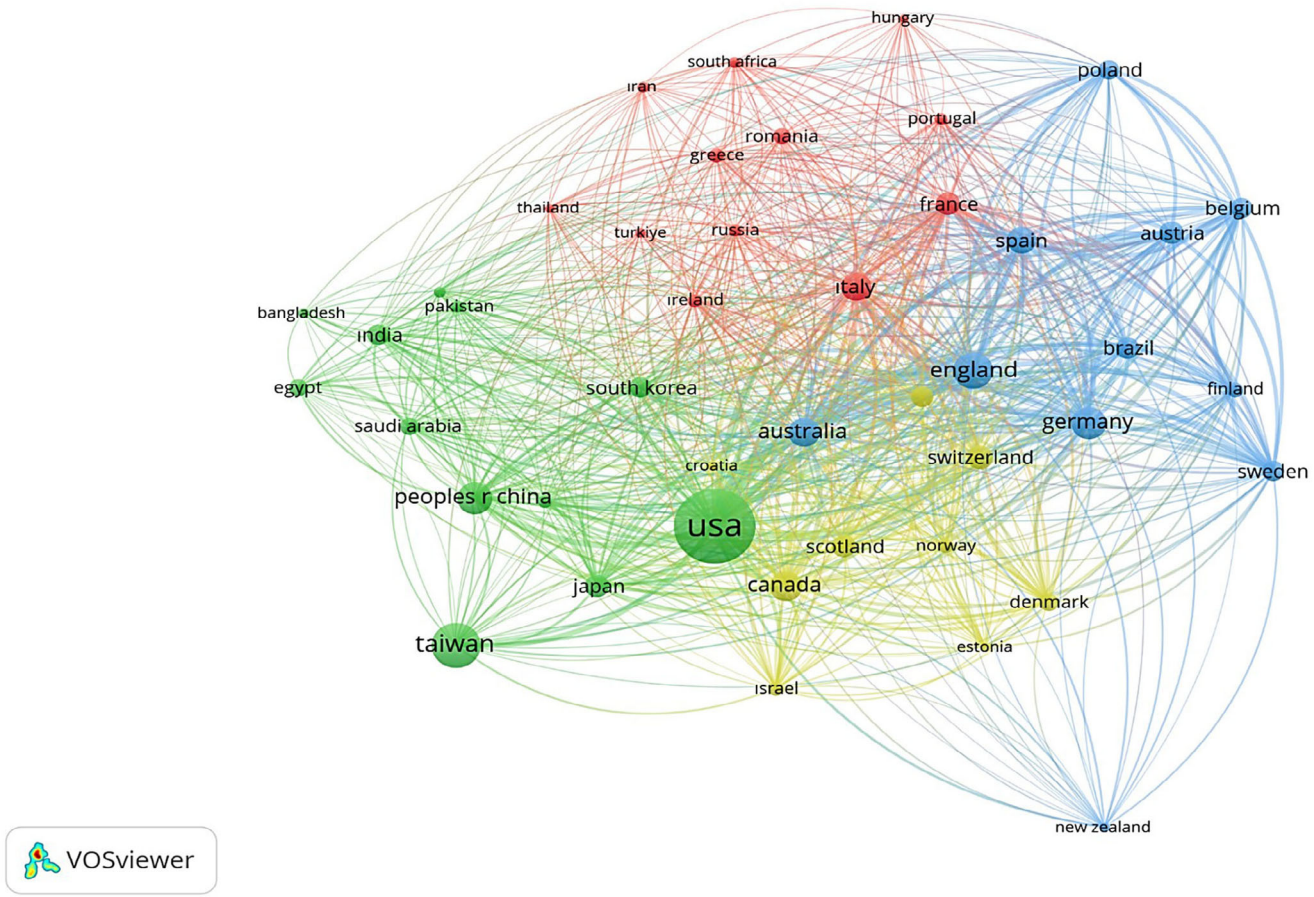
International Collaboration Network Map, where the thickness of the lines represents the strength of collaboration between institutions or countries, and the size of the circles or text indicates the level of international involvement. This map visually highlights the key players in global research and the intensity of their collaborative efforts.

**FIGURE 8:**
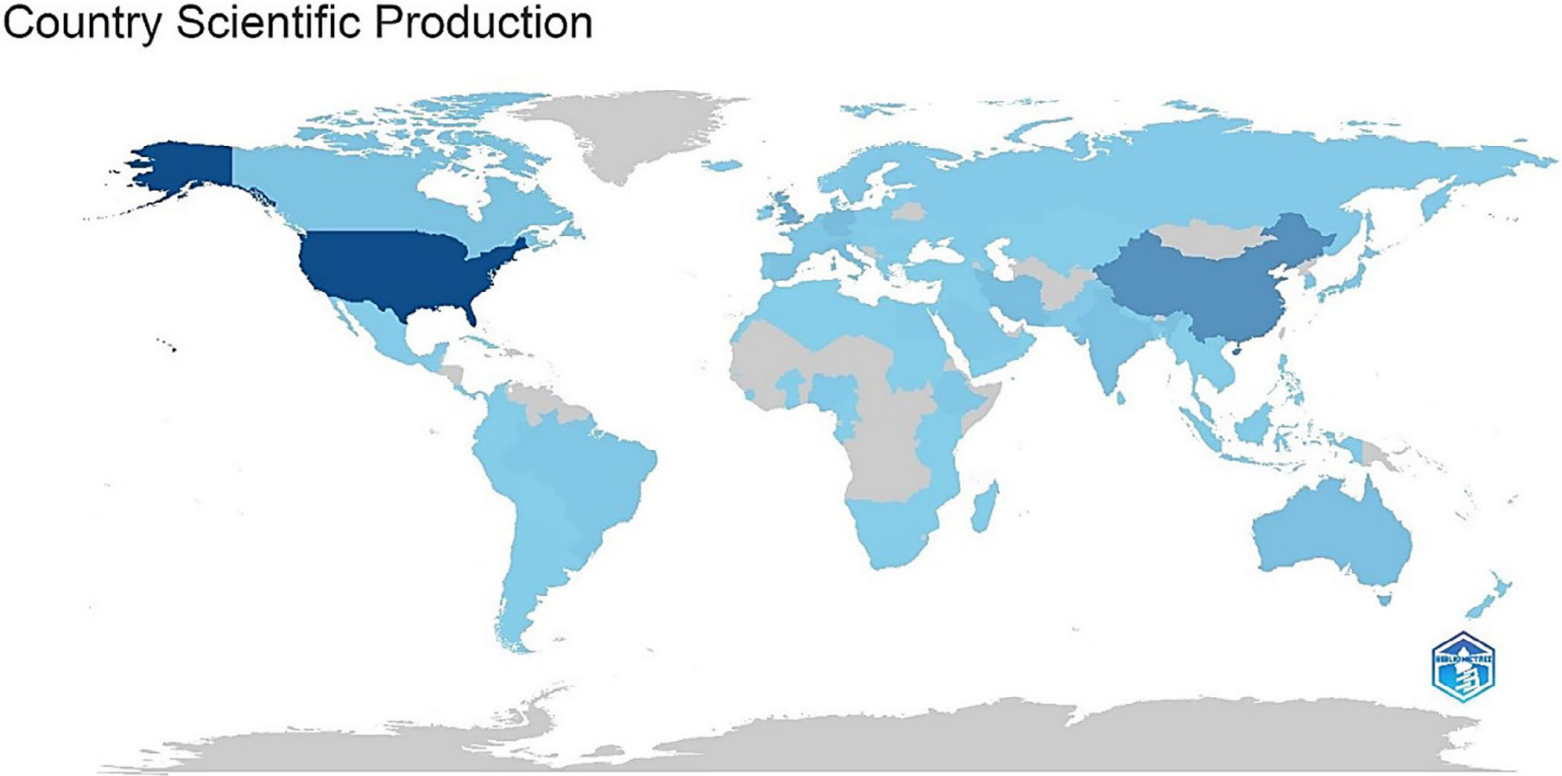
Scientific production density graph of artificial intelligence studies in veterinary medicine by country. In this graph, darker blue regions indicate a higher volume of studies, while grey areas represent countries or regions where no studies have been conducted in this field. The graph visually reflects the global distribution and concentration of research in artificial intelligence within veterinary medicine.

In terms of the number of published articles, the USA emerges as the country with the highest publication rate, contributing 719 articles (51.4%). It is followed by Taiwan with 267 articles (19.1%), the United Kingdom with 184 articles (13.1%), and Germany with 144 articles (10.3%). In addition to these four countries, studies have been published in a total of 140 different countries worldwide. A detailed list of the top 15 countries, each with 70 or more publications, is presented in Table 2. These data illustrate the prevalence of international research in the field of AI‐assisted veterinary medicine and highlight the diverse contributions made by various countries (see Table 2 and Figures 7 and 8).

**TABLE 2:** Countries with at least 20 publications.

Countries/regions	Record count	% of 1400
USA	719	51.36
Taiwan	267	19.07
England	184	13.14
Germany	144	10.29
China	142	10.14
Canada	130	9.29
Australia	123	8.79
Italy	110	7.86
Spain	102	7.29
Switzerland	84	6.00
France	80	5.71
Scotland	80	5.71
Japan	74	5.29
Austria	72	5.14
Brazil	70	5.00

*Note*: Showing 15 out of 140 entries.

Within the scope of this research, US‐based institutions stand out among the prominent contributors. In particular, the US Department of Veterans Affairs accounts for the highest rate of publications at 20.6%, followed closely by the Veterans Health Administration (VHA) with 19.3%. Other notable institutions include Pennsylvania State University with 9.3%, Taipei Veterans General Hospital with 11.6%, and Harvard University with 11.5%. These findings indicate that connections within the United States are predominantly influential in veterinary research related to artificial intelligence. For clarity, only 15 of the total 5576 records are presented in the following table. Details are provided in Table 3 and Figures 7 and 8.

**TABLE 3:** Top affiliations ranking.

Affiliations	Record count	% of 1400
US Department of Veterans Affairs	289	20.64
Veterans Health Administration VHA	270	19.29
Taipei Veterans General Hospital	162	11.57
Harvard University	161	11.50
National Yang Ming Chiao Tung Univ.	156	11.14
University of California System	138	9.86
Harvard Medical School	102	7.29
Stanford University	100	7.14
Massachusetts Institute of Tech. MIT	81	5.79
University System of Ohio	79	5.64
Taichung Veterans General Hospital	71	5.07
University of London	68	4.86
Massachusetts General Hospital	65	4.64
State University System of Florida	62	4.43
University of Texas System	61	4.36

*Note*: Showing 15 out of 5576 entries.

**FIGURE 9:**
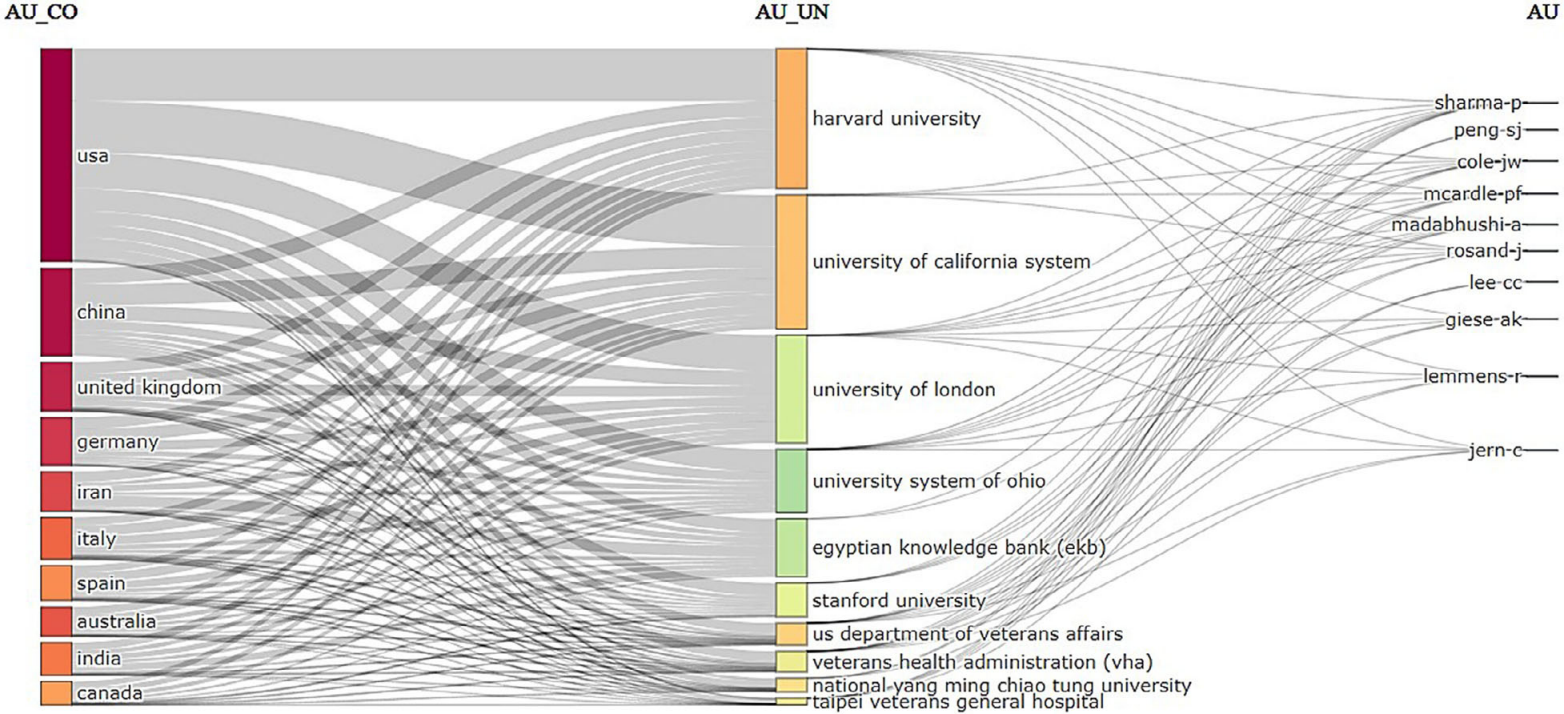
Collaboration graph of artificial intelligence studies in veterinary medicine, illustrating the connections between countries, institutions, and authors. The graph highlights the patterns of research collaboration, showing how different entities work together in advancing AI applications within the field of veterinary medicine.

Figure 9 illustrates the collaboration among countries, institutions, and authors in veterinary artificial intelligence studies. This graph highlights the interactions within the collaboration network, showcasing how different countries, institutions, and authors engage in artificial intelligence research. The lines represent the collaborative efforts, with the thickness of each line indicating the strength of the collaboration. This visualisation allows for the identification of countries and institutions that are actively collaborating, as well as the authors who have made significant contributions to the field. Such an analysis of collaboration can enhance international research partnerships, providing a crucial foundation for the advancement of artificial intelligence applications in veterinary medicine.

In the examinations conducted on Web of Science indexes, it is evident that the majority of publications are classified under the ‘Science Citation Index Expanded (SCI‐Expanded)’ category. Articles in this category constitute 84.6% of the total publications, making it a crucial index that reflects the quality and impact of scientific articles. Following this, the ‘Emerging Sources Citation Index (ESCI)’ ranks second with 9.0%, highlighting its significance in the research landscape by including emerging and developing journals. Lastly, the ‘Social Sciences Citation Index (SSCI)’ holds the third position with 7.7%; this index evaluates articles in the social sciences and demonstrates the social impact of the research. This distribution indicates that studies on the application of artificial intelligence in veterinary medicine prioritise high quality and impact potential in the scientific literature. Detailed data is presented in Table 4.

**TABLE 4:** Web of Science Categories Index.

Web of Science Index	Record count	% of 1400
Science Citation Index Expanded (SCI‐Expanded)	1184	84.57
Emerging Sources Citation Index (ESCI)	126	9.00
Social Sciences Citation Index (SSCI)	108	7.71
Conference Proceedings Citation Index–Science (CPCI‐S)	97	6.93
Book Citation Index–Science (BKCI‐S)	7	0.50

**FIGURE 10:**
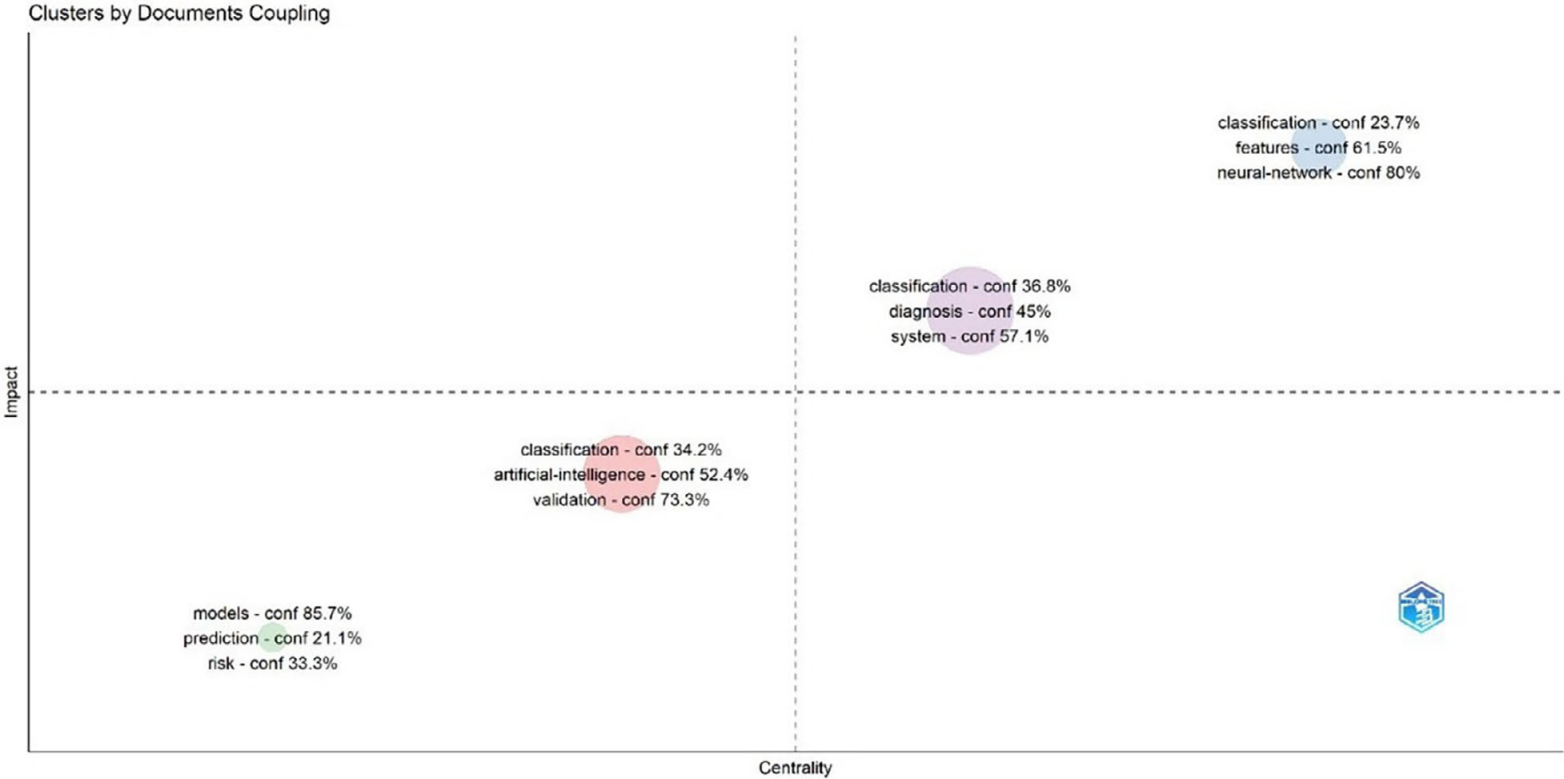
Clusters by documents coupling, depicting the thematic and methodological connections within research areas. This visualisation shows how related documents are grouped based on shared themes and methodologies, offering insights into the interconnections and trends across different research topics.

Figure 10 illustrates the clusters formed by the combination of documents, revealing thematic and methodological connections within research areas. This graph analyses which topics and methods are often cited together across specific documents. The clusters observed indicate how researchers group around particular themes and methodologies and how interactions within these areas are structured.

For example, the figure shows that keywords and concepts such as ‘diagnosis’, ‘classification’, and ‘system’ cluster together, while other concepts like ‘models’, ‘prediction’, and ‘risk’ form distinct groups. Such an analysis can provide valuable insights into the directions of artificial intelligence studies in veterinary medicine, highlight existing gaps in the research area, and ultimately guide future research efforts.

## 4 Discussion and Conclusion

This study demonstrates a global increase in scientific research and publications on artificial intelligence (AI) in veterinary medicine. The primary aim was to identify global trends and clusters within AI research in this field, highlighting specific focus areas and countries where such studies are prevalent. Furthermore, identifying key journals, authors, and notable studies points to potential areas for increased use of animal models in AI research in the future.

The number of bibliometric studies specifically addressing AI applications in veterinary medicine is quite limited. A comprehensive bibliometric analysis in this domain is currently absent from the literature. Existing studies tend to focus on AI applications in general medical contexts, lacking a targeted bibliometric assessment for veterinary medicine. This gap signifies a considerable knowledge deficiency, particularly given the growing significance of AI in this field. Therefore, bibliometric analyses that evaluate research trends, collaborations, and publication dynamics related to AI in veterinary medicine are essential for understanding scientific advancements and guiding future research.

The primary objective of integrating AI into veterinary medicine is to enhance efficiency and, in some cases, improve the quality of care. Research in human medicine has shown that AI and robotic systems can sometimes surpass human surgeons, indicating a similar potential in veterinary procedures. AI has the potential to significantly improve the overall quality of veterinary services by automating various aspects of work management and medical care (INVMA 2024).

In the AI process, several stages are involved, including enriching data with natural language processing (NLP), analysing it with machine learning (ML), and ultimately supporting clinical decision‐making from the clinical data collection phase. Regardless of the sophistication of AI techniques, they should be developed based on clinical problems and directed towards practical clinical applications. A significant portion of the AI literature focuses on the analysis of diagnostic imaging, genetic tests, and electrodiagnostic data, particularly during the diagnosis phase (Jiang et al. 2017). Jha and Topol (2016) suggested that radiologists should utilise AI technologies when analysing extensive volumes of diagnostic images. The IBM Watson system serves as a pioneer in this field, having made significant strides in oncology. For instance, in a cancer study, 99% of Watson's treatment recommendations aligned with physician decisions, and Watson collaborated with Quest Diagnostics to offer Genetic Diagnostic Analysis (Lohr 2016).

Tools like VOSviewer have been employed for detailed analyses of collaboration networks, research trends, and future research topics. Additionally, Bibliometrix and Biblioshiny, free research software operating in R, provide an interactive platform for conducting bibliometric and visual analyses. These methods enhance the accuracy and reliability of research while facilitating better interpretation of the data. The analyses yield a comprehensive view of AI applications in veterinary medicine, supported by diagrams and maps illustrating key research points, processes, and publication dynamics over time (Aria and Cuccurullo 2017).

In a study by Zawacki‐Richter et al. (2019) on AI research, a notable increase in published articles since 2007 was reported. The number of articles rose from just six in 2007 to 23 in 2018. On the other hand, in this study, a total of 1400 articles were analysed, receiving a cumulative 44,700 citations, which means each article averaged 32 citations. The H‐index was determined to be 74, reflecting the impact and prevalence of research in this field. The low number of publications on artificial intelligence, particularly between 1990 and 2015, suggests that artificial intelligence was either not well‐recognised or not sufficiently valued in veterinary medicine during this period. However, since 2019, there has been a noticeable increase in both the number of articles and citations, indicating a growing importance and acceptance of artificial intelligence applications in veterinary medicine. Basran and Appleby (2022) highlighted that despite the recent rise in AI publications, there remain exciting opportunities for AI in veterinary medicine and animal health.

The analysis revealed that significant keywords such as ‘artificial intelligence’, ‘deep learning’, ‘convolutional neural network’, ‘dog’, ‘Covid‐19’, ‘radiology’, and ‘disease’ were frequently used in the studies. These keywords reflect the focal points of AI research in veterinary medicine and the most common topics within these areas. Notably, the rise in studies addressing the effects of Covid‐19 illustrates the pandemic's impact and the necessity for AI in health applications. Here, the role of AI in rapid diagnosis and treatment may become increasingly important in veterinary practice in the future. Sharma et al. (2022) noted AI's crucial role in vaccine design and development research, and the rapid advancement of vaccines against Covid‐19 has underscored AI's transformative potential in this domain. This underscores how AI can significantly accelerate solutions to complex biomedical challenges.

Although specific standards for evaluating the safety and effectiveness of AI systems are currently lacking in regulations, the US Food and Drug Administration (FDA) has initiated guidance studies for assessing AI systems to address this issue (Graham 2016). Zawacki‐Richter et al. (2019) found that the articles examined in their study were published across 104 different journals, indicating the broad impact of AI applications across various disciplines. The analysis results from this study indicated that the fields contributing most significantly in terms of article volume were ‘Veterinary Sciences (11.8%)’, ‘Computer Science (11.6%)’, ‘Science Technology Other Topics (8.1%)’, and ‘Engineering (7.5%)’. The 11% publication rate in veterinary sciences indicates a need for further research in this area. Countries such as the United States (51.4%), Taiwan (19.1%), the United Kingdom (13.1%), and Germany (10.3%) emerged as leading contributors to AI research. Zawacki‐Richter and colleagues (2019) analysed the geographical distribution of articles in their study on artificial intelligence research across all scientific fields. The analysis examined 19 countries. The results show that 50% of the articles came from the United States, China, Taiwan, and Turkey alone. This suggests that AI research is concentrated in these countries.

The prominence of institutions such as the US Department of Veterans Affairs (20.6%), Veterans Health Administration (19.3%), Pennsylvania State University (9.3%), Taipei Veterans General Hospital (11.6%), and Harvard University (11.5%) underscores the US's leadership in this research field. Zawacki‐Richter et al. (2019) noted that most of the examined articles were published by US universities, indicating that academic institutions in the United States significantly drive research in AI. This finding reinforces the US's position as a leader in AI research.

Recent years have seen significant momentum in integrating AI into veterinary medicine. Major events, such as the Cornell Symposium on Artificial Intelligence and Veterinary Medicine (SAVY), and initiatives like the NAVLE (North American Veterinary Licensing Exam) investigating AI involvement, highlight the increasing importance of AI in the veterinary field. Furthermore, the AVMA (American Veterinary Medical Association) has established a task force dedicated to technology and innovation (INVMA 2024).

According to Web of Science indexes, 84.6% of articles are included in the Science Citation Index Expanded (SCI‐Expanded) category, followed by the Emerging Sources Citation Index (ESCI) (9.0%) and Social Sciences Citation Index (SSCI) (7.7%). This distribution indicates that most research is published in high‐impact journals, underscoring the scientific validity of the field.

Given the increasing trend of AI studies in recent years, it is anticipated that such scientific processes will become increasingly significant in veterinary medicine moving forward. Langlotz (2019) posited that radiologists employing AI would outpace those who do not. While there may be no immediate concern regarding the replacement of veterinarians by AI, the potential benefits for both the profession and animal health cannot be overlooked. Appleby and Basran (2022) noted that while there is much to understand about AI, it holds great promise for enhancing veterinary practices.

This research has its limitations. Firstly, bibliometric analysis serves only to identify trends in the literature and does not provide a direct evaluation of data accuracy. Hence, more comprehensive studies are necessary. Conducting similar analyses using various databases could help identify knowledge gaps. Bibliometric analysis aims to examine existing literature and provide a general overview, so the findings warrant deeper investigation through qualitative research. Finally, considering the study's limitations, future research with larger datasets and diverse methodologies is expected to contribute to a more profound understanding of AI literature in veterinary medicine.

In conclusion, this bibliometric analysis delineates delineates global trends and significant studies in the realm of AI in veterinary medicine. It serves as a valuable resource for researchers, policymakers, and industry representatives in the health sector, underscoring the importance of research in this area. Future investigations into AI applications in veterinary medicine could aid in the development of clinical applications and enhance animal health. Additionally, the findings highlight the necessity for further studies in the literature on AI in veterinary medicine. Future research should delve into the effects of AI technologies on animal health and behaviour more comprehensively. Such investigations could contribute to veterinary medicine's evolution and provide innovative solutions in practice. Moreover, comparing AI applications across different countries could foster international collaboration and knowledge sharing. Ultimately, a better understanding of AI's potential in veterinary medicine offers significant opportunities for improving animal health and welfare in the future.

## Author Contributions

All aspects of the article were conducted by SE and OY.

## Ethics Statement

This study is a bibliometric analysis that does not include human or animal experiments; therefore, ethics committee approval is not required.

## Conflicts of Interest

The authors declare no potential conflicts of interest concerning the research, authorship, and/or publication of this article.

## Patient or Public Contribution

Any patients, service users, caregivers, people with lived experience or members of the public are not included in this study.

### Peer Review

The peer review history for this article is available at https://publons.com/publon/10.1002/vms3.70258.

## Data Availability

The data sets generated for this study are available on request to the corresponding author.
